# Randomised-controlled trial of a web-based dietary intervention for patients with type 2 diabetes: changes in health cognitions and glycemic control

**DOI:** 10.1186/s12889-018-5640-1

**Published:** 2018-06-08

**Authors:** Amutha Ramadas, Carina Ka Yee Chan, Brian Oldenburg, Zanariah Hussein, Kia Fatt Quek

**Affiliations:** 1 Jeffrey Cheah School of Medicine and Health Sciences, Monash University Malaysia, Jalan Lagoon Selatan, 47500 Bandar Sunway, Selangor Darul Ehsan Malaysia; 2 School of Psychology, Australian Catholic University, Brisbane, Australia; 3 Melbourne School of Population and Global Health, University of Melbourne, Melbourne, Australia; 4 Department of Medicine, Putrajaya Hospital, Putrajaya, Malaysia

**Keywords:** Diabetes mellitus, type 2, Telemedicine, Diet therapy, Randomised-controlled trial

## Abstract

**Background:**

Increasing prevalence and disease burden has led to an increasing demand of programs and studies focused on dietary and lifestyle habits, and chronic diseases such as type 2 diabetes mellitus (T2DM). We evaluated the effects of a 6-month web-based dietary intervention on Dietary Knowledge, Attitude and Behaviour (DKAB), Dietary Stages of Change (DSOC), fasting blood glucose (FBG) and glycosylated haemoglobin (HbA1c) in patients with uncontrolled HbA1c (> 7.0%) in a randomised-controlled trial (*my*DIDeA) in Malaysia.

**Methods:**

The e-intervention group (*n* = 62) received a 6-month web-delivered intensive dietary intervention while the control group (*n* = 66) continued with their standard hospital care. Outcomes (DKAB and DSOC scores, FBG and HbA1c) were compared at baseline, post-intervention and follow-up.

**Results:**

While both study groups showed improvement in total DKAB score, the margin of improvement in mean DKAB score in e-intervention group was larger than the control group at post-intervention (11.1 ± 0.9 vs. 6.5 ± 9.4,*p* < 0.001) and follow-up (19.8 ± 1.1 vs. 7.6 ± 0.7,*p* < 0.001), as compared to the baseline. Although there was no significant difference between intervention and control arms with respect to DSOC score and glycaemic control, the e-intervention group showed improved DSOC score (199.7 ± 18.2 vs193.3 ± 14.6,*p* = 0.046), FBG (7.9 ± 2.5 mmol/L vs. 8.9 ± 3.9 mmol/L,*p* = 0.015) and HbA1c (8.5 ± 1.8% vs. 9.1 ± 2.0%,*p* = 0.004) at follow-up compared to the baseline, whereas such improvement was not seen in the control group.

**Conclusions:**

Most important impact of *my*DIDeA was on the overall DKAB score. This study is one of the first to demonstrate that an e-intervention can be a feasible method for implementing chronic disease management in developing countries. Concerns such as self-monitoring, length of intervention, intense and individualized intervention, adoption of other domains of Transtheoretical Model and health components, and barriers to change have to be taken into consideration in the development of future intervention programs.

**Trial registration:**

ClinicalTrials.gov NCT01246687.

## Background

Type 2 Diabetes Mellitus (T2DM) is an increasingly important medical and public health issue in many countries, including Malaysia. T2DM is the most common form of diabetes and is characterized by disorders of insulin action and insulin secretion, either of which may be the predominant feature. The latest Malaysian National Health and Morbidity Survey report stated the prevalence of T2DM among adults above 18 years has increased from 11.6% in 2006 to 17.5% in 2015 [1]. The increasing prevalence of diabetes and the resulting disease burden has led to a rising demand for evidence-based programs to improve and evaluate diabetes management, especially in developing countries [2]. Although T2DM could be inherited, modifiable factors such as body composition and nutrition also play important role in the aetiology of T2DM [3].

There is accumulating evidence from a range of different behavioural interventions and delivery methods that have shown promising results in prevention and management of chronic diseases such as T2DM [4]. Behavioural interventions have been proven to assist the management of T2DM and websites were found to be a feasible medium for the delivery of such interventions, though the evidence were mostly from Western cultures and did not focus solely on dietary behaviour [5, 6]. Instead, dietary behaviour has been a component of a number of web-based studies that aimed at preventing T2DM [7–10]. Although limited trials were conducted to test the effectiveness of web-based intervention among T2DM patients, the available evidence has shown an improvement in outcomes for individuals using web-based interventions to increase nutritional knowledge and improve glycaemic control [6, 11]. Web-based interventions have demonstrated some favourable outcomes, provided they are further enhanced with appropriate e-research strategies such as use of e-mail or mobile text message reminder to improve log-in rates and use of local languages in content development [5, 12, 13]. Interactive components with tracking and personalised feedback, as well as peer-support components were also shown to be effective strategies in ensuring the success of web-based intervention for patients with T2DM [6].

The use of Internet has been found to significantly contribute towards improvement of some other health behaviour changes in people with T2DM, especially when the intervention is strengthened with a theoretical framework [14, 15]. Theory and evidence-based behaviour interventions are long hailed to be the ideal approach towards successful health behaviour changes. Although no agreement exists as to the best theories for health promotion purposes, the Transtheorical Model (TTM) [16] has become one of the most popular behaviour change models used in health promotion [17]. The most commonly applied component of TTM is the construct of Stages of Change (SOC), where the participants are classified into one of the five distinct stages; pre-contemplation, contemplation, preparation, action, and maintenance [18]. The TTM and the SOC construct grew on the understanding how people change their behaviour. Few studies have assessed the application of TTM in dietary interventions [14, 19, 20], and more than 75% of studies reviewed by Spencer et al. [14] supported the use of SOC model in dietary interventions. A review of past studies identified various positive impacts of TTM-based dietary interventions, which included reduction of fat consumption, and an increase in the consumption of fruit and vegetables [19]. The evidence is particularly strong in patients with T2DM who received intensive intervention of at least 6 months, and the strong evidence substantiates the effectiveness of self-monitoring among others [20]. Despite the potential, Lee and colleagues (2015) found lack of exploration on the concurrent use of information technology with TTM [21].

To date, there is no published study focused on dietary behaviour change in adults with T2DM via a website-based system. However, dietary modification has been incorporated as a component of a web-based weight-loss program in prevention of T2DM in adults [8]. Calorie count as a part of behaviour e-counselling intervention also significantly reduced the weight of the adults at risk for T2DM [9]. ICAN, a 12-month RCT which tested the efficacy of physical activity and nutrition behaviour changes in improving diabetes control found favourable results as well [10]. Despite the limited evidence, use of Web-based interventions compared to non-web-based interventions showed an improvement in outcomes for individuals using web-based interventions to achieve the specified knowledge and/or behaviour change which include increase in nutritional knowledge and diabetic control [11]. A web-based intervention which gives the flexibility for the participants to log in at their own pace and set personalised goals may yield a better and favourable results than generic and non-web-based interventions.

Taking these factors into consideration, we aimed to evaluate the effects of *my*DIDeA (**M**ala**y**sian **D**ietary **I**ntervention for People with Type 2 **D**iabetes: An **e**-**A**pproach), a 6-month web-based stage-personalised dietary intervention on Dietary Knowledge, Attitude and Behaviour (DKAB), Dietary Stages of Change (DSOC), fasting blood glucose (FBG) and glycosylated haemoglobin (HbA1c) in patients with uncontrolled HbA1c (> 7.0%). We hypothesised *my*DIDeA to result in significant between- and within-group changes in DKAB, DSOC, FBG and HbA1c in our study population.

## Methods

### Study design

**Fig. 1:**
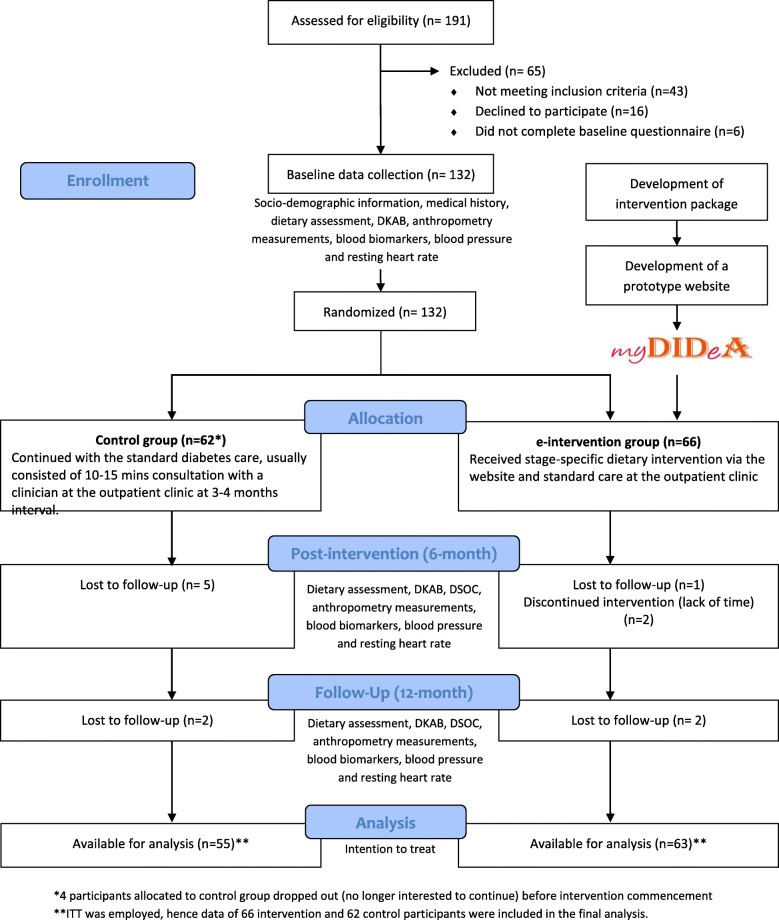
Study flow chart

This was a two-armed multi-centre RCT. The recruitment of subjects, screening and data collections for this study were conducted in three public hospitals in Klang Valley, Malaysia, namely Hospital Putrajaya, Serdang and Selayang. The study was designed according to the recommendations of the CONSORT statement for randomised-controlled trials of nonpharmacological treatment [22] and commenced after obtaining ethical clearance from the Malaysian Ethics Research Committee (NMRR-09-303-3416) and Monash University’s Human Research Ethics Committee (CF09/1583–2,009,000,877). The study flow chart is summarised in Fig. 1. The detailed study protocol has been published previously [23].

### Participant recruitment and randomization process

The eligibility screening (Table 1), recruitment of study participants and data collections were conducted in the outpatient medical and/or diabetes clinics of the three hospitals. Eligible patients, screened by diabetes nurses independent to the study, provided their informed consent. Subsequently, they were randomised to either e-intervention (receiving 6-month web-based dietary intervention in addition to standard care) or control (standard care) groups.

**Table 1:** Eligibility criteria used in the screening of the participants

Inclusion	Exclusion
• Mentally sound men and women who are ≥ 18 years old. • Are literate with a fair command of English and/or Malay languages. • Have access to the Internet at home, work or public place. • Are willing to access the study website at least once every fortnight. • Have been confirmed of having HbA1c of ≥ 7.0%.	• Are pregnant, lactating or intend to become pregnant during the study period. • Are diagnosed with Type 1 Diabetes Mellitus (T1DM) or Gestational Diabetes Mellitus (GDM) • Weigh more than 150% of the desired weight for height. • Have any pre-existing condition compromising the quality of life or ability to participate according to protocol. • Have severe complications (chronic heart disease, cerebrovascular disease, diagnosed HIV/AIDS, cancer, emphysema, chronic liver or kidney disease) that would affect the subjects’ ability to follow the tailored advice. • Are enrolled in other clinical studies. • Have DKAB score more than 50% at baseline.

The allocation sequence was automatically generated based on the order of recruitment. The study researcher took the responsibly of the recruitment process. The study researcher was not blinded of participants’ treatment group, while the attending hospital physicians and other hospital staffs were kept blinded. The screening and recruitment of study participants was conducted for 3 months. However, commencement of the intervention was done as soon as the patient been randomised into one of the study group.

Based on the findings of a previous study [24], a minimum sample of 31 patients was needed in each group to detect a difference in mean behaviour scores between treatment and control groups, with a two-sided alpha of 0.05 and a power of 80%. Based on 30% attrition rate for one year, a minimum of 41 participants were required in each group.

### E-intervention program

The development of the dietary intervention program can be described in the following systematic six-step planning approach – 1) needs assessment; 2) module development; 3) development of strategies according to behavioural theory; 4) detailed lesson plans development; 5) implementation of web-based program and 6) effect and process evaluation of the program.

The literature and existing guidelines for patients with T2DM [25–31] were reviewed as part of the needs assessment to identify specific dietary factors of concern. A module consisting of twelve dietary lesson plans were then developed based on the evidence (Table 2). The content of each of the lesson plans was investigated for its relevance to the local community and tailored to suit local context.

**Table 2:** Sample of the lesson plans

Lesson plan	General recommendations	Intervention objectives	Patient’s objectives
Sugar	• Total free sugar not > 10% unless if the glucose level is under control.	• Educate on various other forms of sugar in food products. • Emphasise on homemade foods with less or no sugar.	• Able to identify and reduce consumption of common food products that are high in hidden sugar.
Fruits & vegetables	• 5–7 servings of fruits and vegetables a day. • Fruits and vegetables to contribute natural micronutrients without the need for supplements.	• Consume fruits and vegetables as whole whenever possible. • Educate and advocate fruits and vegetables as the main source of vitamins and minerals.	• Include fruits and vegetables in main meals or as snacks to achieve the recommended servings per day.
Eating out habit	• Maintain healthy eating outside home.	• Encourage to choose sugar-free or sugar-less meals. • Keep the total calorie intake low when eating out.	• Reduce consumption of artificial flavouring such as sauces, dressings, salt or sugar when eating out. • Learn to pick low calorie foods when dining out.

TTM and SOC constructs [16, 18] were identified to assist with the intervention program design. Program components were developed based on the recommendations, objectives and DSOC. The lesson plans were translated to *Bahasa Malaysia*, the national language of Malaysia and back translated to English. A working plan of the intervention program that would be delivered via the Internet was then developed and a prototype website was piloted in a small group of patients with T2DM (*n* = 30) to assess the acceptability and user-friendliness of the intervention structure and web design.

The staged-tailored recommendations delivered via the study website (*my*DIDeA) were aimed to address the barriers and motivate the participants according to their DSOC. The dietary lesson plans in the intervention package was personalised according to the patients’ DSOC and was expected to improve their DKAB and assist them to progress in their respective DSOC. The improvements in DKAB and progress in DSOC were expected to be reflected in the patients’ FBG and HbA1c.

Only patients recruited into e-intervention group (*n* = 66) were provided access to *my*DIDeA and they were required to login with their unique username and password. Twelve lesson plans were made available to the patients one after another over the period of 6 months, with updates every fortnight. Log-in reminders were sent via e-mail each time *my*DIDeA is updated with new lesson plans and participants were followed-up with text messages if they failed to log-in within three days post-update. The participants were also encouraged to send their queries to the study nutritionist via the website. The effectiveness of the web-delivered intervention program was evaluated via the randomised-controlled trial.

Process evaluation in form of intervention adherence and program reception were conducted at post intervention. On average, each participant logged in at least once for each lesson plan and spent almost 12 min on the site. The participants’ content satisfaction, acceptability, and usability scores were satisfactory. A detailed description of the development and process evaluation of the e-intervention program has been published previously [32].

### Measures

Data were collected at baseline, 6-month post-intervention and 12-months follow-up by independent data collectors. A structured bilingual (English and *Bahasa Malaysia*) questionnaire was used to collect data on socio-demography (age, gender, ethnic group, education, occupation and personal income), family history of diabetes (yes/no), current medication (OHA/insulin/OHA + insulin), duration of diabetes (months/years), self-monitoring of blood glucose (times per week), existing co-morbidities (hypoglycaemia, heart disease, kidney disease, nerve disorder, hypertension and dyslipidaemia), smoking and alcohol drinking habits (yes/no) to describe the study population. Physical activity level was measured using International Physical Activity Questionnaire (IPAQ) [33].

The primary outcomes were changes in DKAB which were measured using a 36-item Dietary Knowledge, Attitude and Behaviour Questionnaire (DKAB-Q), a validated composite assessment of knowledge, attitude and behaviour related to dietary education for people with diabetes in Malaysia. DKAB-Q was developed primarily based on the existing guidelines and recommendations [25–31]. The DKAB-Q consists of three domains – Knowledge (12 points), Attitude (60 points) and Behaviour (12 points), with total maximum score of 84 points. The first domain, Knowledge, measured patients’ understanding of important dietary aspects. The 12 items in this domain were measured using responses of “True”, “False” and “Don’t know”. Each correct response was given one point, whilst incorrect responses as well as “Don’t know” responses were given zero point. The second domain, Attitude, measured the attitude of patients towards diet and diabetes. Five items in this domain were scored using Likert scale responses: strongly agree = 5 to strongly disagree = 1, while 7 reversed items were scored from strongly agree = 1 to strongly disagree = 5. The third domain, Behaviour, measured the dietary behaviour of the patients. Responses to twelve items in this domain were scored as “Yes”, “No” and “Not sure”. The scoring is similar to that of the Knowledge domain. Content and face validity of the instrument have been assessed and DKAB-Q has also shown good internal consistency and test-retest reliability [34].

The impact of the intervention on DSOC, FBG and HbA1c was also evaluated. A 60-item validated five-point Likert-scaled questionnaire was used to determine the DSOC of the study patients. Fasting blood samples for FBG and HbA1c were collected by a trained nurse or phlebotomist from a vein in the arm during patients’ clinic visit. Plasma FBG was measured by UV hexokinase method on automated biochemistry analyser, UNICEL® DXC 800 (Beckman Coulter, Massachusetts, USA), while plasma HbA1c was analysed using the principle of ion-exchange high performance liquid chromatography (HPLC) on the D10 BIORAD system (Biorad Laboratories, Hercules, California, USA). The FBG and HbA1c findings were electronically transferred to patients’ medical records. All enumerators and hospital personnel involved in data collection were blinded to patients’ study groups.

### Analyses

Statistical analysis was undertaken using IBM® SPSS® 20.0 with statistical significance set at *p* = .05. Chi square (χ2) or equivalent was used to determine the association between categorical variables, while independent t-test or equivalent was used to determine the mean differences of continuous variables. Significant differences between and within the study groups at various time points were observed using two-way repeated measures ANCOVA. The evaluation of the intervention was based on an intention-to-treat analysis, applying Last Observation Carried Forward (LOCF) principles [35].

## Results

### Characteristics of the study participants

Participants were 77 male and 51 female patients with T2DM. The recruitment rate among patients who met the study eligibility criteria was 86.5%. Lack of time to be engaged in a trial was the most common reason of refusal. None of the demographic and baseline characteristics of the participants significantly differed between groups (Table 3). The mean age of the study participants was 50.5 years old (*SD* = 10.5). Malay was the largest ethnic group participated in this study (72.6%). More than 60% of the study participants had tertiary level education and were employed full-time with a mean personal income of MYR 5166 (*SD* = 3816) per month (approximately USD1,223 (*SD* = 903)).

**Table 3:** Demography and baseline characteristics of the study participants

		e-intervention	control	Total
		n = 66	n = 62	N (%)
Demographic characteristics				
Age		49.6 (10.7)	51.5 (10.3)	50.5 (10.5)
Gender	% male	62.1	75.8	60.2
Ethnic group	% Malay	69.7	75.8	72.6
Education	% tertiary	62.1	54.8	61.7
Occupation	% employed	68.2	54.8	61.7
Personal income (MYR)		4837 (2571)	4813 (4672)	5166 (3816)
Medical condition				
Diabetes duration (months)		111.1 (106.3)	81.8 (69.9)	96.9 (91.3)
Family history of diabetes	% yes	84.8	82.3	83.6
Diabetes medication	OHA	48.5	50.0	49.3
	Insulin only	10.6	3.2	7.0
	OHA + insulin	37.9	35.5	36.7
	Unknown	3.0	11.3	7.0
Self-monitor blood glucose	Daily	9.1	14.5	11.7
	4–6 times/week	13.6	1.6	1.7
	1–3 times/week	71.2	53.2	62.5
	Less than once a week	3.0	6.5	4.7
	No	15.2	24.2	19.5
Self-reported clinical history	Hypoglycaemia	7.6	3.2	5.5
	Heart disease	6.1	14.5	10.2
	Kidney disease	1.5	0.0	0.8
	Nerve disorder	3.0	8.1	5.5
	Hypertension	54.5	48.4	53.1
	Dyslipidaemia	48.5	43.5	46.1
Lifestyle				
Total MET/week^a,b^		879.0 (269.5)	620.3 (335.7)	733.0 (213.2)
Physical activity level	Low	37.9	50.0	43.8
	Moderate	39.4	30.6	35.2
	High	22.7	19.4	21.0
Smoking	% yes	13.6	17.7	15.6
Alcohol drinking	% yes	10.6	4.8	7.8
*Diet*				
Total DKAB score		34.2 (5.2)	33.7 (5.5)	33.9 (5.4)
Total DSOC score		193.3 (14.6)	191.2 (16.2)	192.3 (15.4)
*Glucose control*				
Fasting blood glucose (mmol/L)		8.9 (3.9)	8.3 (2.9)	8.6 (3.5)
HbA1c (%)		9.1 (2.0)	8.9 (1.9)	9.0 (2.0)

Data are presented as means (SD) and percentages, or as ^a^medians (SE) for skewed data

^b^Assessed based on International Physical Activity Questionnaire (IPAQ)

Two-tailed independent t-test (or Mann Whitney Rank Test) and chi-square analysis (or Fisher Exact Test) were performed between study groups. None of the analysis were significant at *p* < 0.05

On average, study participants had been diagnosed with T2DM 8 years previously. A large percentage (83.1%) of them had family history of diabetes. Almost half of the study participants were being treated with oral hypoglycaemic agent, with equal distribution in types of diabetes treatment in both groups. Slightly more than 80% of them were self-monitoring their blood glucose at home, with the majority monitoring it one to three times a week. Based on the self-reported medical history, hypertension (53.1%), dyslipidaemia (46.1%) and previous history of heart diseases (10.2%) were the most common co-morbidities among the participants.

Physical activity was determined by the total MET per week [36]. The total MET/week was 733.0 (*SD* = 213.2). Almost 44% of the participants had low physical activity level, while only 21.0% of them reported to be engaged in high level of physical activity. Only 15.6 and 7.8% of the participants were current smokers and drinkers, respectively.

The mean DKAB score was 33.9 (*SD* = 5.4), which was about 57% of total score of 60 points. The mean DSOC score was 192.3 points (*SD* = 15.4) out of a total possible score of 300 points. The mean FBG and HbA1c were 8.6 mmol/L (*SD* = 3.5) and 9.0% (*SD* = 2.0), respectively.

### DKAB score

**Fig. 2:**
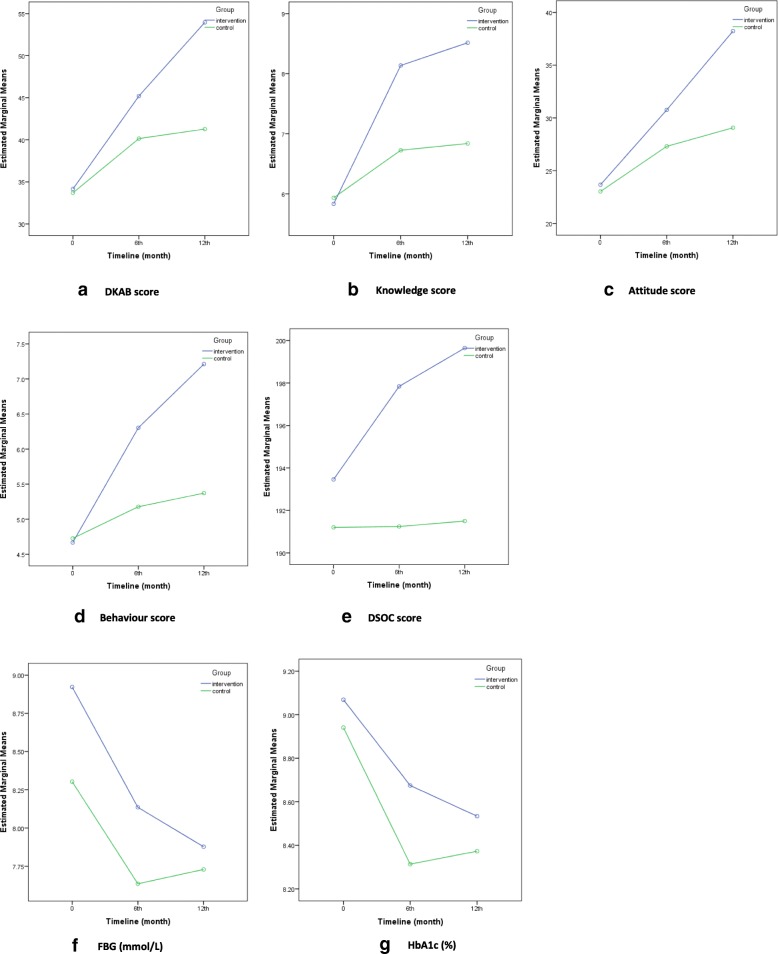
Changes in mean DKAB score and its sub-domains (**a**-**d**), (**e**) DSOC score, (**f**) FBG(mmol/L) and (**g**) HbA1c(%) across data collection points

An overall significant difference in total DKAB score between timelines was found in the e-intervention (*F* = 244.212, *p* < 0.001, ƞ2 = 0.790) and in the control (*F* = 62.453, *p* < 0.001, ƞ2 = 0.676) groups (Table 4). Significant difference was found between the study groups (*F* = 26.818, *p* < 0.001, ƞ2 = 0.175) and there was also a significant interaction between the study groups and increasing scores across the timeline (*F* = 53.059, *p* < 0.001, ƞ2 = 0.296). Figure 2(a) further confirms the rapid increase in the score in the e-intervention group compared to the control group.

**Table 4:** Within and between study groups comparison at various data collection points

		Timeline (month)			Within study group			Between study groups			Interaction (Timeline^*^study group)		
		0	6th	12th	F	P	ƞ^2^	F	P	ƞ^2^	F	P	ƞ^2^
Total DKAB score	e-intervention	34.2 (5.2)	45.2 (8.0)	54.0 (8.7)	244.212	< 0.001^**^	0.790	26.818	< 0.001^**^	0.175	53.059	< 0.001^**^	0.296
	Control	33.7 (5.5)	40.2 (9.9)	41.3 (7.7)	62.453	< 0.001^**^	0.676						
*Knowledge score*	e-intervention	5.8 (2.1)	8.1 (2.2)	8.5 (2.0)	102.738	< 0.001^**^	0.612	12.027	< 0.001^**^	0.087	23.824	< 0.001^**^	0.159
	Control	5.9 (1.7)	6.7 (1.6)	6.8 (1.5)	13.540	< 0.001^**^	0.182						
*Attitude score*	e-intervention	23.7 (4.1)	30.8 (6.2)	38.2 (6.7)	221.521	< 0.001^**^	0.773	21.680	< 0.001^**^	0.147	38.478	< 0.001^**^	0.234
	Control	23.0 (4.3)	27.3 (8.6)	29.1 (6.7)	38.318	< 0.001^**^	0.618						
*Behaviour score*	e-intervention	4.7 (1.8)	6.3 (2.1)	7.2 (2.0)	103.180	< 0.001^**^	0.614	9.076	0.003^*^	0.067	27.205	< 0.001^**^	0.178
	Control	4.7 (2.1)	5.2 (2.1)	5.4 (2.1)	6.168	0.004^*^	0.092						
DSOC score	e-intervention	193.3 (14.6)	197.5 (16.7)	199.7 (18.2)	3.305	0.046^*^	0.049	7.552	0.007^*^	0.057	1.488	0.229	0.012
	Control	191.2 (16.2)	191.2 (17.1)	191.5 (15.1)	0.008	0.992	0.000						
FBG (mmol/L)	e-intervention	8.9 (3.9)	8.1 (2.7)	7.9 (2.5)	6.054	0.015^*^	0.085	0.899	0.345	0.007	0.591	0.453	0.005
	Control	8.3 (2.9)	7.6 (2.6)	7.7 (2.6)	2.501	0.117	0.039						
HbA1c (%)	e-intervention	9.1 (2.0)	8.7 (1.9)	8.5 (1.8)	8.334	0.004^*^	0.114	0.433	0.511	0.003	0.793	0.383	0.006
	Control	8.9 (1.9)	8.3 (2.1)	8.4 (2.2)	10.934	0.001^*^	0.152						

Data expressed as mean (SD); e-intervention (n = 66) and control (n = 62)

^*^significant at *p* < 0.05; ^**^significant at *p* < 0.001

Similar to total DKAB score, diabetes-related dietary knowledge, attitude and behaviour domain scores were significantly different across the timeline in both groups. Significant interactions were found between the knowledge (*F* = 23.824, *p* < 0.001, ƞ2 = 0.159, attitude (*F* = 38.478, *p* < 0.001, ƞ2 = 0.234), behaviour (*F* = 27.205, *p* < 0.001, ƞ2 = 0.178) domain scores and study groups. The increase in the scores levelled after the 6th month and overall, the participants in e-intervention group had higher score at all data collection points (Fig. 2(b), (c) and (d)).

### DSOC score

In contrast to DKAB score, there was an overall significant difference in DSOC score between timelines only among the e-intervention participants (*F* = 3.305, *p* = 0.046, ƞ2 = 0.049) and there was a significant difference in score between study groups (*F* = 7.552, *p* = 0.007, ƞ2 = 0.057). Figure 2(e) presents the patterns of DSOC score change.

### Glycaemic control

Although decreasing trends of mean FBG(mmol/L) and HbA1c(%) can be seen in both groups (Fig. 2(f) and (g)), the within group analyses showed a significant decrease in FBG among e-intervention group participants at follow-up compared to post-intervention (*F* = 6.054, *p* = 0.015, ƞ2 = 0.057). The mean HbA1c of the e-intervention group has significantly decreased by 0.5% at post-intervention (*F* = 8.334, *p* = 0.004, ƞ2 = 0.114) and 1.5% at 12th month follow-up (*F* = 10.934, *p* = 0.001, ƞ2 = 0.152) as compared to the baseline. However, no significant difference in the group*timeline interactions, changes in FBG and HbA1c between groups and within the control group were found (Table 4).

## Discussion

Continuous education through the *my*DIDeA website on various lesson plans has shown a positive impact on the DKAB. The intervention program successfully assisted the participants to achieve a better DKAB score, possibly through matching motivational readiness and accelerating the learning process.

Recommendations based on the current DSOC probably gave more valid and feasible suggestions to the patients, leading to improvements of the DKAB scores. The e-intervention group scored much higher DKAB score than the control suggesting that receiving additional education through *my*DIDeA could result in improved dietary health cognitions and behaviours. Similar to the total DKAB score, the knowledge and attitude score of participants in both groups have increased, but those in the e-intervention group have shown a higher margin of increase in score compared to the control group. However, only the e-intervention group has shown significant improvement in the dietary behaviour score at post-intervention and follow-up.

The encouraging improvement in knowledge score of the e-intervention participants showed that the intervention was successful in conveying the necessary dietary information, comprehension and skill of the participants. The intervention program also improved the attitude score of the participants, which reflected on positive reaction to the knowledge provided. The improvement of behaviour score in the e-intervention group, further emphasized the success of *my*DIDeA and it showed that the web-based dietary intervention program can propagate positive change in dietary behaviour. A more comprehensive dietary education program that addressed barriers such as lack of support from family and health services, time management and dietary myths [37–39] could result in an even better dietary behaviour change. A successful program could also take into consideration factors such as meal planning, diet quality, self-monitoring, dietary self-efficacy, social support and time management [40–43], and deliver it through trained professionals such as dieticians or nutritionists functioning as part of multidisciplinary teams [43–45].

The DSOC score itself is a continuous measure of the participants’ SOC and it was used to determine participants’ SOC for each dietary lesson plan, and the recommendations given were strictly based on the score obtained. The e-intervention group had slightly higher DSOC score compared to control group at the baseline. As the study progressed, the DSOC of e-intervention participants exhibited greater increase than the control group. The increase in DSOC suggests that the intervention was effective in improving the participants’ dietary behaviour, as a higher DSOC score reflects a more advanced readiness to change.

The participants showed higher SOC probably because the majority of them were educated with access to the Internet. It is highly likely that they were aware of the importance of dietary changes in T2DM and trying to change their dietary behaviour. However, without much guidance and intervention it might not be that easy to propagate further from the action stage. It has been highlighted that T2DM patients’ SOC varies according to dietary areas and within the dietary habits [46] and the SOC construct will be more useful when used together with other TTM measures such as decisional balance and self-efficacy in developing a more well-rounded intervention program [47].

There is some level of evidence to suggest that the e-intervention through *my*DIDeA website was able to help the participants achieve better glycaemic control. Although there is no quality data to support the clinical efficacy of online dietary intervention in improving glycaemic control among people with T2DM, past reviews have suggested improvements in FBG, HbA1c and diabetes knowledge, besides reducing blood pressure, body weight, waist circumference and need for medication following an intervention program [29, 48–50]. The reduction in FBG and HbA1c in *my*DIDeA is comparable to the findings from other web-based lifestyle interventions, which reported a reduction of 0.78 mmol/L of FBG and 0.19 to 0.59% of HbA1c in patients with T2DM [10, 51]. Website-based self-monitoring interventions have also suggested improvement in both glycaemic control and lipid markers [52, 53].

The decreasing trend in HbA1c and FBG in *my*DIDeA is encouraging, and it can be anticipated that with longer duration of follow-up and regular reinforcement, better and clinically significant glycaemic control could be achieved.

### Strengths and limitations

Those with T2DM may be extremely receptive towards improving their diet and physical activity behaviours and this offers the researcher a captive or “teachable moment” to promote behaviour change which may ultimately prevent or delay the onset of diabetes-related complications. Besides, the probability of success in intervention or programs related to dietary behaviour modification increases as the interventional strategies more aptly address the diversity of racial, ethnic, cultural, linguistic, religious and social factors in a community. Thus, dietary interventions that are personalised and modified to suit the local context such as *my*DIDeA, would have much greater impact than those that only promote the general guidelines.

The increasing government’s effort to popularize high-speed broadband and Internet in general, has already resulted in an increasing Internet penetration in Malaysia. While the use of Internet to educate patients is a fairly new area of clinical and research interest in developing countries such as Malaysia, findings from this study have provided support for such education program to be incorporated into the existing healthcare system. This study’s findings can be seen as representative of other low- and middle income countries (LMICs) which share similar socio-economic background as Malaysia.

The *my*DIDeA is one of the few web-delivered dietary interventions for patients with chronic disease. Being a web-based study, the intervention was flexible to the participants’ availability. Besides, the reinforcement with e-mail and subsequent text message reminders has been helpful to keep the compliance rate high, with average frequency of log-in at 1.26 (*SD* = 0.16) and duration spent at 11.93 (*SD* = 2.90) per lesson plan [32]. This has opened up more avenues for future web-based studies to focus on dietary behaviours.

The intervention program has merged four widely accepted guidelines and recommendations for patients with T2DM in Malaysia [25, 26, 28, 31]. This has enabled the researchers to diversify the content of the intervention by incorporating the international recommendations and local guidelines into one program. Most of the dietary factors specified in the guidelines have been explored and included in the intervention program without being too technical. The content of the intervention has also been adapted to suit the local culture. For example, we included various types of local dishes with culturally and religiously-sensitive options in the module. This culturally adapted intervention can be more widely implemented in other states within the country or in neighbouring regions with a similar culture.

Most of the available diabetes Knowledge, Attitude and Behaviour (KAB) or Knowledge, Attitude and Practice (KAP) do not focus solely on diet. While this made the outcome comparison to be more difficult, it also showed the importance of administering a validated questionnaire solely to assess the dietary education for people with diabetes. The DKAB-Q which was developed closely with *my*DIDeA’s dietary module, measured the impact of the intervention on the primary aspects of dietary KAB.

The intervention program development had only utilised the SOC in the intervention design. Other constructs of TTM such as process of change could be used, and these constructs have been shown to be more effective than using just SOC alone. The web system only managed to track the login frequency and time spent on the website. Another feature such as ‘e-Mail the Nutritionist’ was not extensively used.

Based on the feedback received from the participants, the two weeks’ gap between modules was considered too long. It was initially meant for the patients to adapt to the recommendations and make necessary changes before the next module and not to make them feel rushed. As the study was conducted for a long period (12 months) among patients with uncontrolled diabetes, there is a possibility for changes to be made to the standard treatment or medication given by the attending physician. The trial was also not blinded, with patients and investigators knowing the treatment allocation. While these may potentially be confounding variables, the randomised study design and blinding of attending hospital physicians are expected to eliminate or control these possible confounders.

Patients were recruited in the clinical setting and all data collections were carried out in the clinics when they attend follow-up treatment. The participants were not required to come to the hospital specifically for data collection purpose, unless they have missed their appointment. The aim was to reduce any additional burden to the patients and this might be associated with better compliance to the intervention program.

The selection of patients with DKAB score of less than 50% and HbA1c of more than 7% meant that only those in dire need of dietary education to improve their glycaemic control were included in this RCT. This offered a teachable moment for the researchers, and detection of significant changes in important outcome measures.

### Future direction

While the anxiety of the participants in using a new system is understandable, future researchers could prepare the participants to use the system by offering short workshop on web usage at the beginning of the intervention. As the two-week gap between the module updates was deemed too long, future studies following the similar style to *my*DIDeA could opt for a shorter time interval between updates.

*my*DIDeA has demonstrated that the intake of fruits and vegetables were below the national recommendation level. Emphasis can be given on fruits and vegetables in future research, as these are major sources of micronutrients and beneficial phytochemicals, and will add value to a dietary intervention. Web-based interventions should also include an option for the patients to input their blood sugar levels, dietary intake or other measures to encourage interactivity and self-monitoring behaviour.

Narrowing the gap between scientific evidence and practice is an emerging priority in health research, particularly in developing countries [54]. The outcomes from this study could be used to strengthen the diabetes management initiatives. While the use of computers may have some limitations, mobile phones tend to have a better penetration rate in these countries. Hence, e-interventions such as *my*DIDeA can be adopted for mobile use to reach more communities.

## Conclusion

In summary, *my*DIDeA was a successful intervention program to improve the overall DKAB score, aided by the improvement in the knowledge and attitude sub-domains. However, other issues such as addressing the barriers, shortening the study duration, making use of other health components, inclusion of self-monitoring and more intense and individualized intervention, would have likely made a difference in the behavioural aspect. The study did find an increasing trend in DSOC score among intervention participants though the increase was not statistically significant. Excluding patients in the action or maintenance stage, and including only those in the pre-contemplation or contemplation stage could be a good option to focus the intervention on those who absolutely need it. Such measures would allow the researchers to investigate the possible movement in SOC and a better dietary practice and glycaemic control could have achieved. Besides, other domains of SOC such as process of change and decisional balance should also be taken into consideration in the development of the intervention.

## Abbreviations

DKAB: Dietary Knowledge, Attitude and Behaviour
DSOC: Dietary Stages of Change
FBG: fasting blood glucose
HbA1c: glycosylated haemoglobin
*my*DIDeA: Malaysian Dietary Intervention for People with Type 2 Diabetes: An e-Approach
SOC: Stages of Change
T2DM: Type 2 Diabetes Mellitus
TTM: Transtheoretical Model
